# Functional optical design of thickness-optimized transparent conductive dielectric-metal-dielectric plasmonic structure

**DOI:** 10.1038/s41598-022-13038-y

**Published:** 2022-05-25

**Authors:** Çağlar Çetinkaya, Erman Çokduygulular, Feyza Güzelçimen, Barış Kınacı

**Affiliations:** 1 Physics Department, Faculty of Science, Istanbul University, 34134 Istanbul, Turkey; 2 Department of Engineering Sciences, Faculty of Engineering, Istanbul University-Cerrahpaşa, 34320 Istanbul, Turkey

**Keywords:** Photonic crystals, Optics and photonics, Materials for optics

## Abstract

Dielectric/metal/dielectric plasmonic transparent structures play an important role in tailoring the high-optical performance of various optoelectronic devices. Though these structures are in significant demand in applications, including modification of the optical properties, average visible transmittance (AVT) and colour render index (CRI) and correlated colour temperature (CCT), obtaining optimal ones require precise thickness optimization. The overall objective of this study is the estimation of the optimal design concept of MoO_3_/Ag/WO_3_ (10/d_Ag_/d_WO3_ nm) plasmonic structure. To explore the proper use in optoelectronic devices, we are motivated to conduct a rigorous optical evaluation on the thickness of layers. Having calculated optical characteristics and achieved the highest AVT of 97.3% for d_Ag_ = 4 nm and d_WO3_ = 6 nm by the transfer matrix method, it is quite possible to offer the potential of the structure acting as a transparent contact. Notably, the colour coordinates of the structure are x = 0.3110 and y = 0.3271, namely, it attributes very close to the Planckian locus. This superior colour performance displays that MoO_3_/Ag/WO_3_ shall undergo rapid development in neutral-colour windows and LED technologies. Structure with d_Ag_ = 6 nm and d_WO3_ = 16 nm exhibits the highest CRI of 98.58, thus identifying an optimal structure that can be integrated into LED lighting applications and imaging technologies. Besides the colour of structure with d_Ag_ = 4 nm and d_WO3_ = 8 nm is equal for D65 Standard Illuminant, the study reports that the range of CCTs are between 5000 and 6500 K. This optimization makes the structure employable as a near-daylight broadband illuminant. The study emphasizes that optimal MoO_3_/Ag/WO_3_ plasmonic structures can be used effectively to boost optoelectronic devices' performance.

## Introduction

Photonics has hitherto been adopted as a fabless technology for generating and harnessing light. Its integration into optoelectronic applications has been pioneered to improve photonics-based innovative device designs by tailoring to optimise their electrical and optical characteristics. These designs are being made to precisely estimate the optimal parameters of the active regions and the devices' selective conduction layers, electrodes, and integrated photonic structures. Notably, the critical significance of visualisation transparent electrodes should be underscored right here, such that these electrodes comprise design parameters considered in the analysis of electrical and optical behaviour of the multilayer-based semi-transparent (ST) optoelectronic devices.

Thanks to the high average visible transmittance (AVT) and electrical conductivity, the use of Gallium-doped Zinc Oxide (GZO), Fluorine-doped Tin Oxide (FTO), and Indium-doped Tin Oxide (ITO) material systems as transparent electrodes has extensively prevailed over a narrow range of optoelectronic applications. However, their integration into flexible devices is inconvenient because they contain rare-earth compounds, high mechanical resistance, low flexibility properties, aggressive techniques of deposits for organic materials and brittleness^1–3^. The increased prices and poor mechanical properties of ITO, FTO, and GZO are undesirable for flexible and novel optoelectronic devices^4^. Besides, while the sheet resistance (${R}_{sh}$) for ITO is about 15 $\Omega {sq}^{-1}$, ${R}_{s}$ below 5.75 $\Omega {sq}^{-1}$ can be obtained in DMD structures where Ag is used^5,6^.

Graphene, carbon nanotube, and conductive polymer-based systems developed as substitute materials to ITO, FTO, and GZO have been limited uses cause of their high cost and low conductivity^5,7–9^. The metal mesh-based transparent electrodes have possessed easy-to-manufacture, inexpensive and abundant, whereas they have exhibited optical turbidity and poor optical performance by reflecting and scattering light in metal mesh patterns^1,10–14^. Contrarily to this case is principally inappropriate for high-resolution display technologies. In addition, considering the varying thicknesses of ITO, FTO and GZO in the range of 100–300 nm, DMD structures that can be designed much thinner below 50 nm are promising for felxiable and lightweight optoelectronics. During recent years, Dielectric-Metal-Dielectric (DMD) structures that are free from drawbacks mentioned above and highly applicable have highlighted their use as alternative materials for transparent electrodes and prevailed after all.

High transparency in DMDs is achieved by sandwiching a thin metallic film of optimal thickness between two anti-reflective dielectrics. Light interference and resonance effect can be observed within the resulting structure by selecting the dielectric material consisting of oxide layers with a high refractive index^3,15,16^. DMDs are also called plasmonic structures in which the effect of surface plasmons (SP) formed by the excitation of conduction electrons in the region close to the metal surfaces is observed^17–20^. By estimating the SP effect, applications including optical cases in which the light energy will be captured, concentrated or transmitted in DMDs can be created as various functional designs^17^.

Moreover, DMD transparent contact structures remain critical components of various optoelectronic devices such as sensors, displays, LEDs and solar cells, as they feature high transparency and conductivity, low haze, excellent flexibility and gerat compatibility with different substrates^6,14,15,21,22^. Since DMDs, compared to other transparent contact structures, can be easily modelled at the microscale through shadow mask lithography, they are pretty functional, possessing the merits of easy fabrication^10,23–25^.

In addition to the metallic layer, the choice of dielectric material in DMD designs is crucial for optoelectronic devices integration and modification of optical properties. Metals with high free electron density provide high conductivity and generate an opposite and uniformly induced electric field within the metallic layer. Free electrons will be driven into surfaces until the build-up electric field produced by the surface charges cancels applied within the metals. Therefore, this process will adversely affect the propagation of light in the metal and reduce the transparency of the structure. This physical process works in reverse; electrons are bound to atoms and can only move around atoms. This results in extremely low conductivity. In this case, due to the polarisation of the atoms under the applied electric field, a relatively weak induced electric field will form in the dielectric material. This field is not strong enough to suppress the outer field. This physical process facilitates the passage of light through the structure, and an increase in the transparency of the structure occurs^14^. Therefore, the optimisation of the dielectric layer is highly influential on the optical characteristics of the DMD structure.

The fact that metals are highly reflective in the visible region (VR) generates a troublesome trade-off between electrical conductivity and optical transmittance, and that is why a major design problem can be presented for metal-based transparent electrodes. Therefore, the material and thickness value of the metallic layer should be noted as dramatically important optimisation parameters for a functional DMD design. Due to the nature of transparent electrodes, with the requirement for low reflectivity, photon absorption and reflection within the electrode structure should be carefully tuned to allow sufficient light to pass through^10,26^.

In addition to optical optimisation, the dielectrics in the DMD structure can also affect the electrical performance of the optoelectronic device they are integrated into. Transition metal oxides (TMOs), which have high transparency in the VR, work functions ranging from 2 to 7 eV, and wideband gaps, can be used as a dielectric layer in DMDs^27^. Thus, TMOs can also act as electron transport layer (ETL) and hole transport layer (HTL) in structures where the DMD system is integrated^10^. In this way, DMD structures carry transparent contact potential and play an influential role in adjusting electrical properties with appropriate dielectric selection^28^. In addition, TMOs can also act as an anti-reflective coating in silicon and organic semiconductor-based optoelectronic devices with appropriate refractive index and band gap values.

To date, a considerable number of studies have notedly addressed DMD structures designed with the use of MoO_3_ and WO_3_ among various TMOs^6,10,20,22,29^. Because DMD designs containing MoO_3_ and WO_3_ have shown extremely promising performances in screens, tunable mirrors, energy-saving intelligent windows, and electrochromic devices with dynamic variability, whose transparency, colour or other optical properties can be adjusted in response to applied potential^22,30–34^. In addition, unlike the symmetrical DMD structures MoO_3_/Ag/MoO_3_ and WO_3_/Ag/WO_3_ DMD structures, the non-symmetrical MoO_3_/Ag/WO_3_ structure offers a more effective use. While MoO_3_ as the inner dielectric layer exhibits a good HTL layer performance in optoelectronic devices, WO_3_, which is used as the outer dielectric, attracts much attention, especially among electrochromic materials due to its chemical stability, strong adhesion to various substrates and high colouring efficiency^6,28,29,35–38^.

The DMD-based transparent contact structure should be designed based on the applicability potential of the optoelectronic device it is integrated into. For example, for semi-transparent solar cells to be used in window applications, the DMD structure should have high AVT values ​​and colour coordinates close to the Planckian locus or D65^10,22^. In the science of color, the Planckian locus is the line that follows the color coordinates of the temperatures of black bodies. In other words, it is the path that the color of an incandescent black body follows in any chromaticity space as its temperature changes^39^. The DMD structure to be used in LEDs designed for high-resolutiondisplay technologies should also have a neutral colour and have a very high colour render index (CRI)^29,40^. At the same time, correlated colour temperature (CCT) is another critical parameter for these structures. These parameters are directly or indirectly related to the thickness of the very thin metallic layer and the lower and upper dielectric materials. In addition, the wavelength dependence of the refractive indices and extinction coefficients of the selected materials for DMD design is also essential.

**Figure 1:**
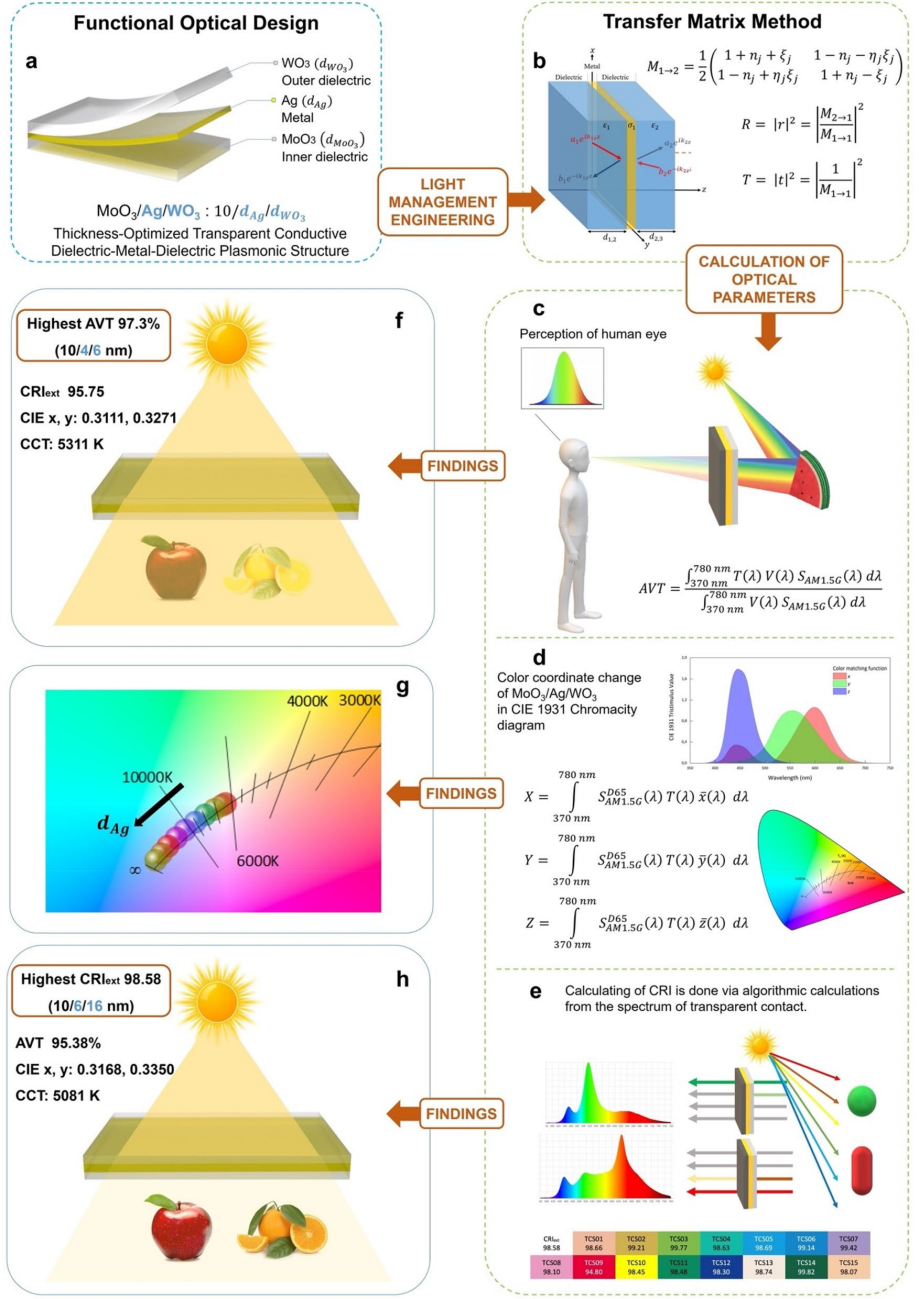
Functional optical design of transparent conductive plasmonic structure and its optical properties. (**a**) Schematic representation of MoO_3_/Ag/WoO_3_ transparent conductive plasmonic structure. (**b**) Transfer Matrix Method used in the calculation of optical characteristics and its application in Dielectric/Metal/Dielectric plasmonic structures. Calculation of optical parameters: (**c**) Evaluation of average visible transmittance calculated based on human eye perception. (**d**) Determination of CIE 1931 color coordinates. (**e)** Obtaining of color rendering index and test color samples. Findings in MoO_3_/Ag/WoO_3_ transparent conductive plasmonic structure, which is optimally designed by evaluating on the basis of optical parameters: (**f)** Highest Average Visible Transmittance. (**g**) Change of CIE 1931 color coordinates. (**h**) Highest color rendering index.

The present study sought to address the potential for use as a multi-purpose of MoO_3_/Ag/WO_3_ DMD transparent contact in various optoelectronic applications like semi-transparent solar cells, window applications, high-resolution LED technologies, lighting and, display technologies involving specific colour applications. By this means, we have functionally realized the contact design with optimal values of optical parameters such as AVT, CRI, CCT and colour coordinates by thickness optimizing of MoO_3_/Ag/WO_3_ in the mentioned application areas. We have employed the conventional Transfer Matrix Method (TMM) in calculations and made a detailed evaluation of the layer thickness dependent data. For the optimal MoO_3_/Ag/WO_3_ (10/${d}_{Ag}$/${d}_{{WO}_{3}}$ nm) design, we have expressed the AVT, CRI and CCT from the findings of transfer matrix method. By its optical characteristic extracted, the current structure may be exhibited as a feasible transparent contact structure with a great colour rendering property. In this study, the methodology followed in the functional optical design of the transparent conductive plasmonic structure and the findings in its optical properties are given in Fig. 1.

## Result and discussion

It is evident that DMD based transparent contact structures have low resistance value (< 10 $\Omega {sq}^{-1}$), efficient charge injecting, and transmittance of more than 70%, particularly in the VR^16,41^. MoO_3_, which acts as an internal dielectric in the MoO_3_/Ag/WO_3_ transparent contact design, has relatively high hole mobility and high transparency in the VR^10,42^. That is why the potential of using MoO_3_ among TCOs with high work function in designing high-performance optoelectronic devices stands out. The use of MoO_3_, which provides a hole injection into active layers and acts as an anti-reflective layer for reflective metals such as Ag and Au, which have a high refractive index^43,44^, as HTL has become a well-established approach in inverted structures^10,45–47^. For optimal electrical performance, the thickness of MoO_3_ affected the electrical parameters of the devices is preferred around 10 nm^28^.

On the other hand, Ag is frequently preferred as a metal layer with a low absorption coefficient and high electrical conductivity than other common metals in DMD structures^48,49^. When an effective cap layer of WO_3_ is put to use due to its high refractive index and prevention of Ag oxidation^50,51^, the radiation loss from the surface plasmon in the Ag layer can be suppressed, and thus the thickness of WO_3_ (${d}_{{WO}_{3}}$) and the permeability of the DMD structure can be modified^6,52^.

By arguments of the sort mentioned above, a detailed evaluation of optical parameters such as AVT, extended CRI (CRI_ext_), CCT, and colour coordinates of the MoO_3_/Ag/WO_3_ transparent contact designed within the scope of the study has been provided based on the thickness change of the metal and WO_3_ layers. For MoO_3_/Ag/WO_3_ (10/${d}_{Ag}$/${d}_{{WO}_{3}}$ nm), the theoretical reflectance, transmittance and absorption spectra were calculated by ranging of 0–20 nm, the effective range where the actual physical changes can be observed. Subsequently, optical parameters of the structures were estimated from the spectra obtained by TMM.

## Evaluation on AVT values of MoO_3_/Ag/WO_3_ structure

**Figure 2:**
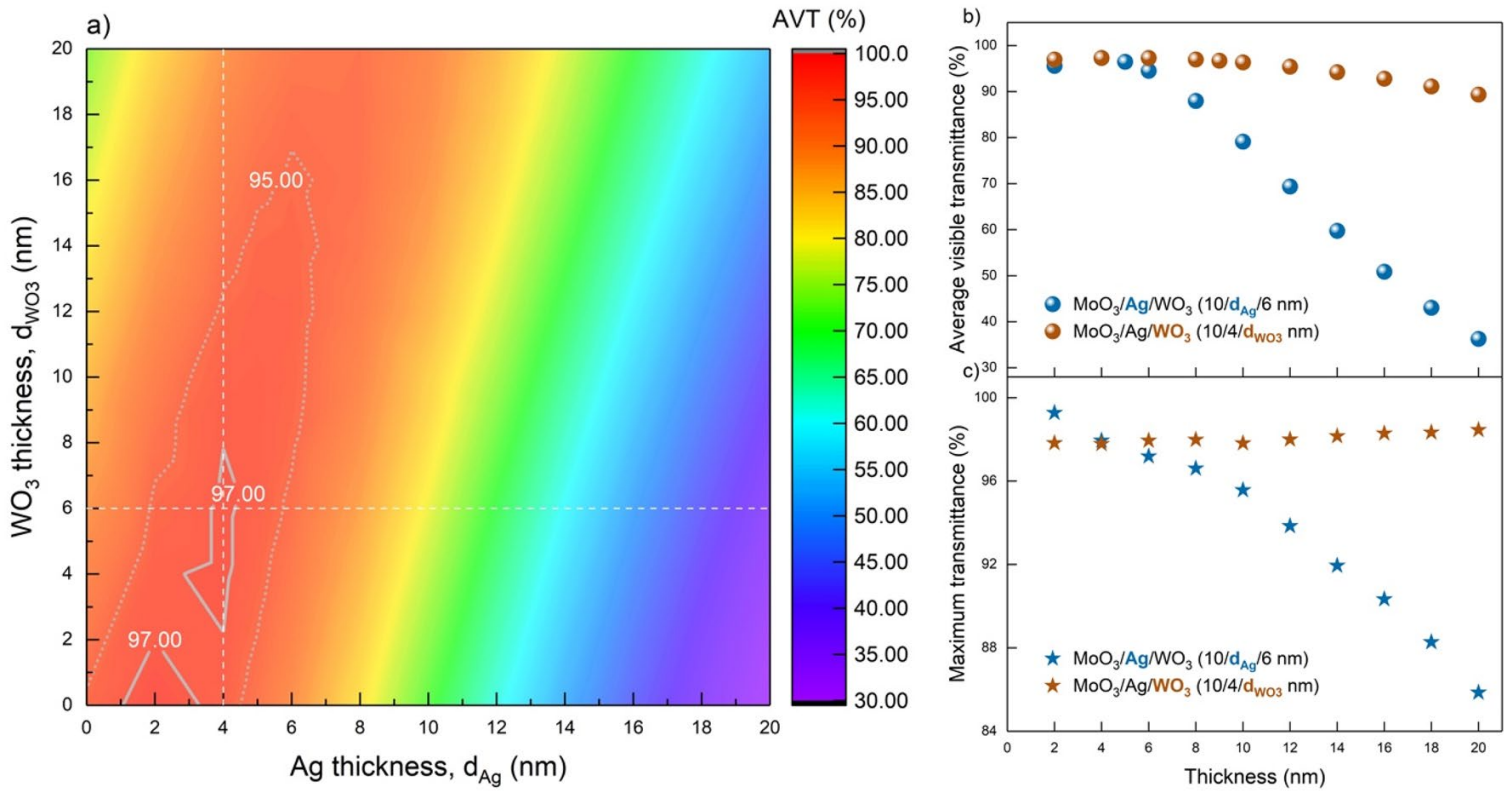
Variation of AVT and transparency depending on the thickness of the transparent contact structure. (**a**) Mapping the AVT variation of the structure according to $d_{Ag}$ and $d_{{WO_{3} }}$ values for MoO_3_/Ag/WO_3_ (10/$d_{Ag}$/$d_{{WO_{3} }}$ nm). (**b**) AVT and, (**c**) $T_{max}$ values for structures of 10/4/$d_{{WO_{3} }}$ nm and 10/$d_{Ag}$/6 nm. Areas with solid grey lines and grey dashed lines have AVTs greater than 97% and 95%, respectively. The point where the white horizontal and vertical lines intersect gives the maximum AVT. It should be noted here that Ag is a determining layer for both AVT and maximum transparency, and outer dielectric WO_3_ acts as a waveguide. The encircling effect on the electromagnetic wave of WO_3_ causes an increment in maximum transparency with the increase of $d_{{WO_{3} }}$. Even the $d_{Ag}$ and $d_{{WO_{3} }}$ values of 20 nm, AVT is greater than 25% which is the lower limit for window applications.

The first investigation for optimal MoO_3_/Ag/WO_3_ (10/${d}_{Ag}$/${d}_{{WO}_{3}}$ nm) design was demonstrated depending on AVT. The ${d}_{Ag}$ and ${d}_{{WO}_{3}}$ values dependence of the structure's associated AVT variation are exhibited in Fig. 2a. By varying ${d}_{Ag}$ and ${d}_{{WO}_{3}}$ values in the range of 0–20 nm, the minimum AVT of the current structure was 32.48%, namely, it is widened to even higher levels than those of the lower limit (25%) for window applications. Based on our analysis, the MoO_3_/Ag/WO_3_ structure is a convenient transparent contact.

Increasing ${d}_{{WO}_{3}}$ at a constant value of ${d}_{Ag}$ has little or no overall effect on AVT levels and the highest AVT was achieved for ${d}_{Ag}$=4 nm and ${d}_{{WO}_{3}}$=6 nm. On the contrary, the increase in ${d}_{Ag}$ considerably affects on AVT. Particularly for greater than 8 nm of ${d}_{Ag}$, the AVT tends to drop below 80%. Therefore, it is identified that Ag in the transparent contact structure exerts a more dominant role over the AVT. The present case can be explained by the reflection characteristic of Ag known in the VR, the electric field provided by the high free electron density, and the SP effect. As seen in AVT distributions, a significant linearity is observed between ${d}_{Ag}$ and ${d}_{{WO}_{3}}$ (Fig. 2a). When this relationship between ${d}_{Ag}$ and ${d}_{{WO}_{3}}$ is maintained in the MoO_3_/Ag/WO_3_ structure, the same AVTs can be obtained. In addition, for an electrical evaluation, in DMD structures in which Ag is a metal layer in the literature, with an increase of ${d}_{Ag}$ from 2 to 12 nm, resistivity and ${R}_{sh}$ decrease from 5.97 × 10^–5^ cm$\Omega$ to 0.97 × 10^–5^ cm$\Omega$ and from 18.66 $\Omega {sq}^{-1}$ to 2.31 $\Omega {sq}^{-1},$ respectively^53^. Therefore, increasing ${d}_{Ag}$ significantly reduces AVT and increases conductivity.

In the AVT mapping of MoO_3_/Ag/WO_3_ (10/${d}_{Ag}$/${d}_{{WO}_{3}}$ nm), the improvement in AVT with the increase of ${d}_{{WO}_{3}}$, especially for a specific ${d}_{Ag}$ larger than 6 nm, is due to the anti-reflection property of WO_3_ with the waveguide effect. Because it acts as the anti-reflection layer of WO_3_ for Ag with a high refraction index [1, 1:35, 1:36]. For a more effective evaluation of the anti-reflection feature, the change in the reflection spectra with the change of ${d}_{{WO}_{3}}$ for 8 nm, 12 nm, 16 nm and 20 nm values of ${d}_{Ag}$ is given in Supplementary Fig. 4. In addition, a more effective anti-reflection feature with ${d}_{{WO}_{3}}$ change by calculating the average reflection over AM1.5G for MoO_3_/Ag/WO_3_ (10/${d}_{Ag}$/${d}_{{WO}_{3}}$ nm) is presented in Supplementary Fig. 5. AR_AM1.5G_ is highly dependent on ${d}_{Ag}$, but for a given Ag layer thicker than 6 nm, the reflection property of the structure is reduced by increasing ${d}_{{WO}_{3}}$.

CRI_ext_, CIE x and y, CCT, maximum transmittance (T_max_) and wavelength (${\lambda }_{max}^{T}$) at T_max_, AVTs according to the MoO_3_/Ag/WO_3_ transparent contact structures change are presented in Table 1a. AVTs, estimated in this study, inside the areas with solid grey lines and grey dashed lines are greater than 97% and 95%, respectively (Fig. 2a). The lowest and highest AVTs of the DMD structure are 32.48% and 97.3% with ${d}_{Ag}$=4 nm, ${d}_{{WO}_{3}}$=6 nm and with ${d}_{Ag}$=20 nm, ${d}_{{WO}_{3}}$=2 nm respectively.

**Table 1:** Optical parameters of the transparent contact structure at different thickness values.

	MoO_3_/Ag/WO_3_ ($d_{Ag}$/$d_{{WO_{3} }}$)	AVT (%)	CRI_ext_	CIE $x$	CIE $y$	CCT (K)	$T_{max}$(%)	$\lambda_{max}^{T}$(nm)
(a)	**4/6**	**97.3**	95.75	0.3111	0.3271	5311	97.95	566.6
	2/1	**97**	94.73	0.3118	0.3262	5273	> 99	709
	2/20	**84.58**	96.67	0.3259	0.3359	4729	> 99	968
	20/20	**53.66**	95.96	0.2483	0.2697	11,528	87.43	435
	20/2	**32.48**	95.82	0.2437	0.2488	14,821	79.68	384.2
(b)	**6/16**	95.38	**98.58**	0.3168	0.3350	5081	97.09	582.2
	14/12	68.26	**98.23**	0.2672	0.2882	8507	94.58	433
	14/6	59.74	**97.16**	0.2632	0.2793	9222	91.95	406.2
	4/6	97.3	**95.75**	0.3111	0.3271	5311	97.95	566.6

CRI_ext_, CIE x and y, CCT, $T_{max}$ and $\lambda_{max}^{T}$ values for MoO_3_/Ag/WO_3_ (10/$d_{Ag}$/$d_{{WO_{3} }}$ nm) transparent contact structure for certain thicknesses according to various (a) AVT and (b) CRI_ext_ distributions. The $d_{Ag}$/$d_{{WO_{3} }}$ values given in bold are the optimal values with the highest AVT in the a section and, the highest CRI_ext_ in the b section.

When both Table 1a and Fig. 2a are interpreted, an increment in AVTs occurs with increasing ${d}_{{WO}_{3}}$, particularly for ${d}_{Ag}$ thicker than 10 nm. For example, in the MoO_3_/Ag/WO_3_ (10/20/20 nm), where the lowest AVT of 32.48% is observed, the AVT improves to 53.66% with the increase of ${d}_{{WO}_{3}}$ to 20 nm. This is since the transparency reduced by the strong electric field and the plasmonic effect observed due to surface charges, especially in thicker metals, is improved with a thicker outer dielectric layer^6,52^. This effect is not dominant for the thin metal layer, and a reduction occurs with ${d}_{{WO}_{3}}$. In the high AVT region, while the AVT is 97% at ${d}_{Ag}$=2 nm and ${d}_{{WO}_{3}}$=1 nm, the AVT decreases to 84.58% and contrarily ${d}_{{WO}_{3}}$ increases to 20 nm.

In the case of ${d}_{Ag}$=2 nm, ${T}_{max}$ is above 99% for ${d}_{{WO}_{3}}$=1 and 20 nm. Since the AVT has a maximum at 550 nm and is evaluated on AM1.5G, the deviation of ${\lambda }_{max}^{T}$ from 550 nm reduces the AVTs even in the case of ${T}_{max}$>99. Therefore, AVT is not at its maximum value in high-transparency structures, and ${\lambda }_{max}^{T}$ values are considerably greater than 550 nm for these structures. With increasing ${d}_{{WO}_{3}}$ from 2 to 20 nm, ${\lambda }_{max}^{T}$ increases from 709 to 968 nm, and contrarily AVT decreases from 96.9% to 84.58%.

As seen in the AVT distribution of the MoO_3_/Ag/WO_3_ (10/${d}_{Ag}$/${d}_{{WO}_{3}}$ nm), the intersection of the horizontal and vertical dashed lines corresponds to the values of ${d}_{Ag}$=4 nm and ${d}_{{WO}_{3}}$=6 nm (Fig. 2a). The changes in AVT and ${T}_{max}$ along these horizontal and vertical lines for thicknesses varying in the range of 0–20 nm are introduced in Fig. 2b and c, respectively.

**Figure 3:**
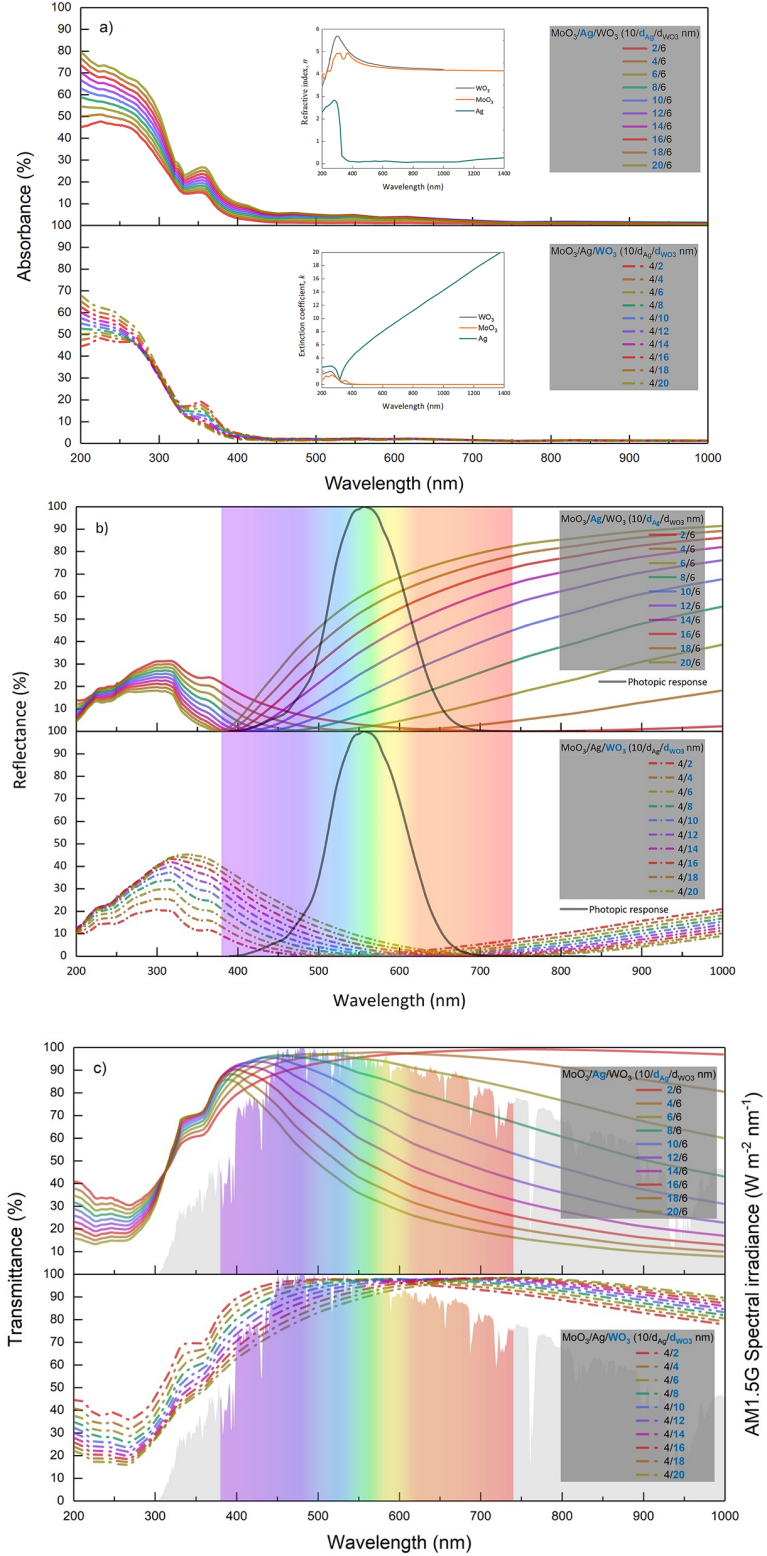
Optical spectra of the transparent contact structure. Variations of (**a**) absorption, (**b**) reflectance and (**c**) transmittance spectra dependent on $$ d_{Ag}$$ and $$d_{{WO_{3} }}$$ of MoO_3_/Ag/WO_3_ (10/$$d_{Ag}$$/$$d_{{WO_{3} }}$$ nm) transparent contact structure. In a, wavelength dependent the refractive index ($$n$$) changes and extinction coefficient ($$k_{ex}$$) of MoO_3_, WO_3_, and Ag are presented. It should be noted that the difference between the n values of MoO_3_ and WO_3_ drastically changes the $$n$$ distribution in the structure and allows for ultra-thin designs that allow easier modification of thickness control and optical characteristics. This enables the design of lighter and more flexible structures with the desired optical properties by using less material. The $$k_{ex}$$ values in the VR for MoO_3_ and WO_3_ are very close to zero, allowing the evaluation of the direct trade-off between transmittance and reflectance without absorption. In b, the visible spectrum and the wavelength range from which the AVTs are calculated are given. In c, the photonic response of the human eye and the VR are presented as enveloped by AM1.5G. When the maximum value of the transparency coincides with the AM1.5G maximum, the highest AVT should be expected in the structure. In addition, the photonic response of human eye determines a wavelength range in the AVT calculations, and an evaluation should be made in this range.

In a transparent contact structure, AVTs are more affected by the change of ${d}_{Ag}$ than ${T}_{max}$. This optical characteristic can be understood when the absorption, reflectance and transmittance spectra of the MoO_3_/Ag/WO_3_ (Fig. 3a–c, respectively) are calculated by TMM according to the ${d}_{Ag}$ ${d}_{{WO}_{3}}$ changes are examined. The AVTs are more sensitive to the change of ${d}_{Ag}$ than ${T}_{max}$ due to the significant increment in the reflection spectrum in the infrared region (IR) and VR. Because the absorption of the structure in this region is deficient and at wavelengths greater than 400 nm, the reflectance and transmittance characteristics are in exchange, as well as their sum is equal to 1. Therefore, increasing reflectance values by ${d}_{Ag}$ in VR decreases the transmittance values. Since AVT values ​​are evaluated directly based on human eye perception, they ​​decrease drastically compared to maximum transmittance.

When the absorption spectra given in Fig. 3a are examined, it is seen that the MoO_3_/Ag/WO_3_ transparent contact has very high absorption in the ultraviolet (UV) region. This is due to the known absorption characteristics of TMOs such as MoO_3_ and WO_3_ in the UV region. In the wavelength region more significant than 400 nm, MoO_3_ and WO_3_ have no absorption, and this characteristic indicates that the MoO_3_/Ag/WO_3_ system is a perfect transparent contact in VR.

**Figure 4:**
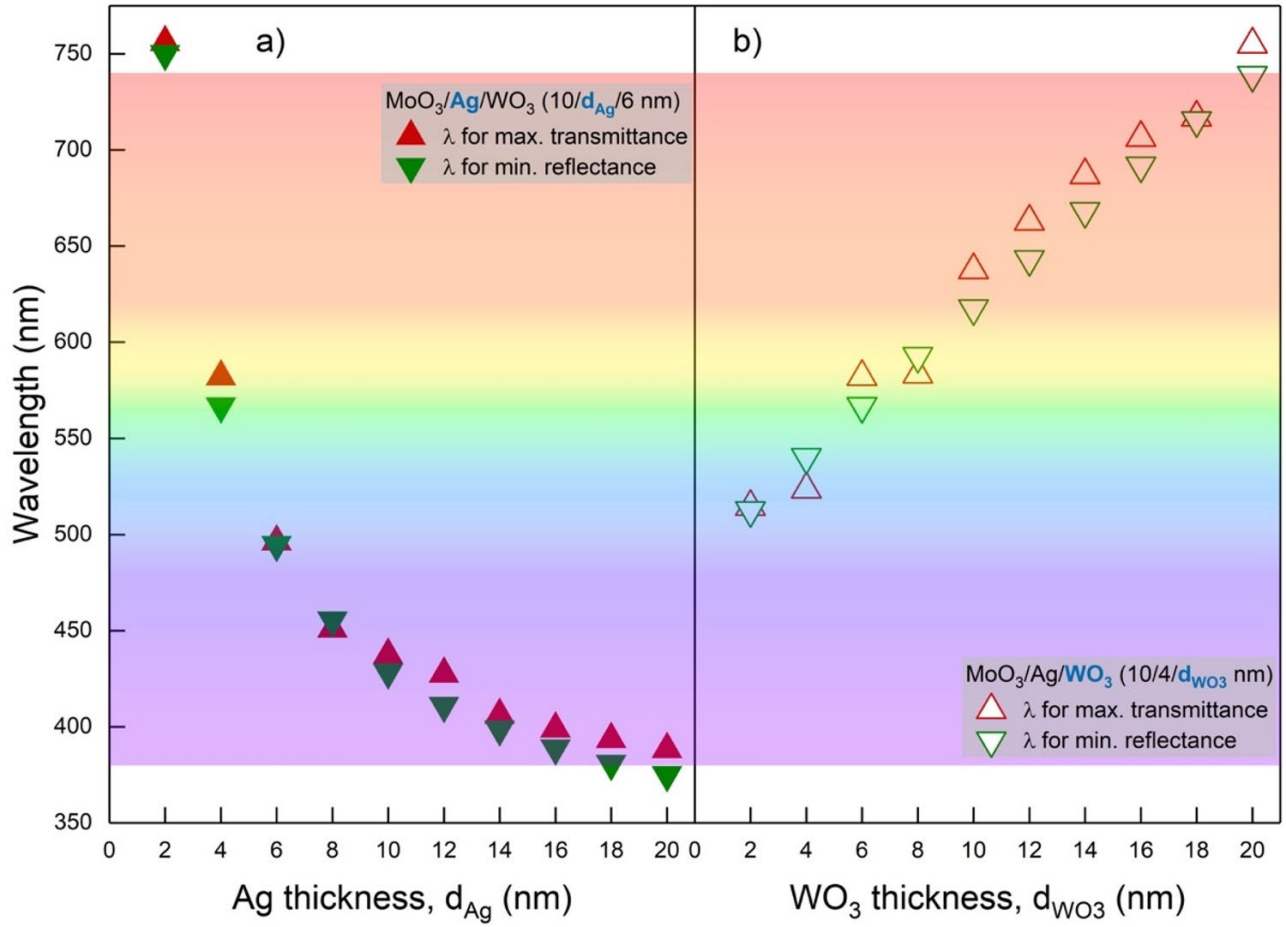
Maximum transmittance and reflectance values of the transparent contact structure. Wavelength changes of minimum reflectance and maximum transmittance corresponding to (**a**) $d_{Ag}$ and (**b**) $d_{{WO_{3} }}$ values in MoO_3_/Ag/WO_3_ (10/$d_{Ag}$/$d_{{WO_{3} }}$ nm) transparent contact structure. Particularly, structures with minimum reflection at wavelengths close to 550 nm can be used as antireflection in various optoelectronic devices. It can be designed that a transparent or an anti-reflection contact system is optimized for lower wavelengths with $d_{Ag}$ and for larger wavelengths with $d_{{WO_{3} }}$.

When the reflectance spectra given in Fig. 3b are examined, the wavelength value with the minimum reflection shifts to a lower wavelength with the increase of ${d}_{Ag}$, while it shifts to higher wavelength values with the increase of ${d}_{{WO}_{3}}$. In addition, the increase in both ${d}_{Ag}$ and ${d}_{{WO}_{3}}$ narrows the distribution of reflectance spectra. This behaviour observed in the reflection characteristic also manifests itself in the transmittance spectra, especially for the wavelength region with no absorption. The variation of wavelength values, at which minimum reflection and maximum transmittance are obtained, concerning ${d}_{Ag}$ and ${d}_{{WO}_{3}}$, are given in Fig. 4a and b, respectively.

The minimum reflectance and maximum transmittance values for ${d}_{Ag}$= 4 nm and ${d}_{{WO}_{3}}$= 6 nm are around 550 nm. Considering that AM1.5G and human eye perception have a maximum value at 550 nm, MoO_3_/Ag/WO_3_ system designed with ${d}_{Ag}$= 4 nm and ${d}_{{WO}_{3}}$=6 nm has the potential to both transparent contact with high AVT for semi-transparent optoelectronic devices and an anti-reflection system for photovoltaic-based optoelectronic devices. In addition, due to the absorption characteristics of TMO’s in the UV region, there is a slight difference between the wavelengths belonging to the maximum transmittance and minimum reflectance for the same ${d}_{Ag}$ and ${d}_{{WO}_{3}}$.

**Figure 5:**
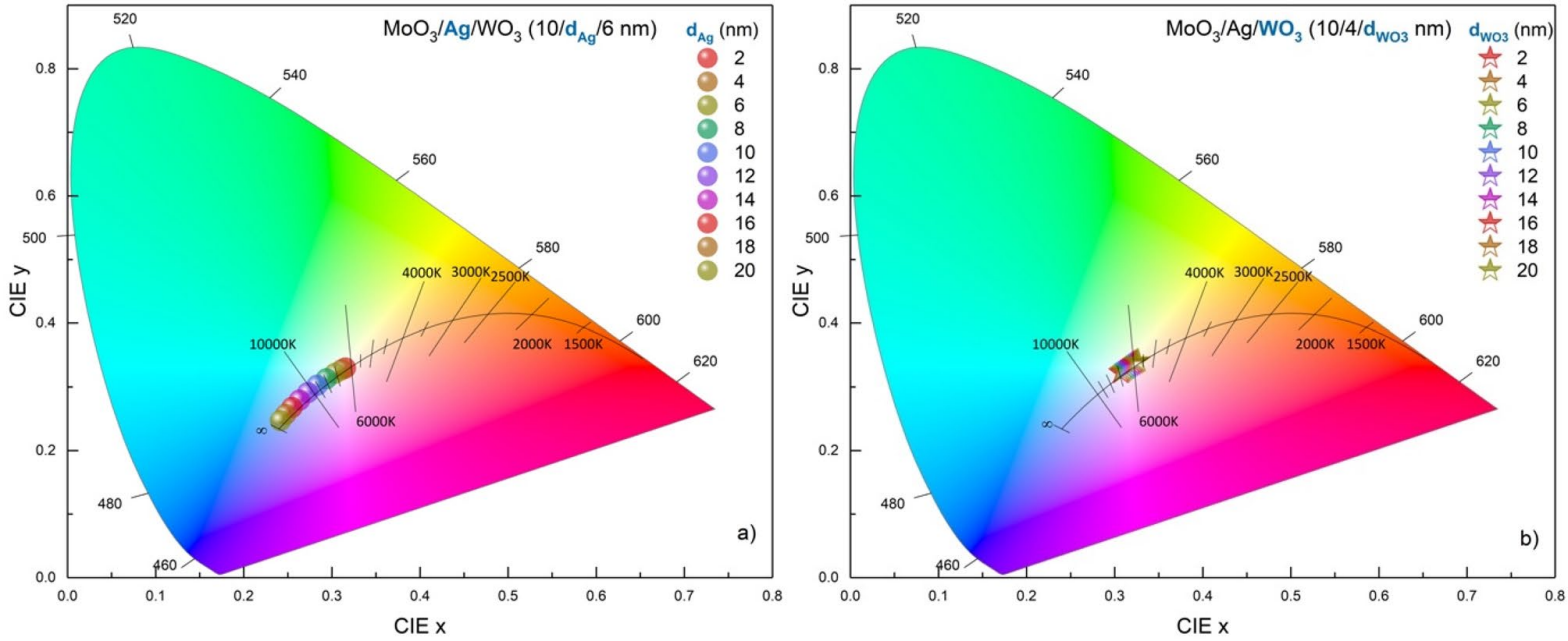
Colour coordinates of the transparent contact structure. In CIE 1931 Chromacity diagram, representation of colour coordinate changes corresponding to $d_{Ag}$ of MoO_3_/Ag/WO_3_ (10/$d_{Ag}$/6 nm) transparent contact structure. The black line in the diagrams shows the Planckian locus values for different CCTs. In the colour coordinates of the contact, a blue shift on the Planckian locus occurs with the $d_{Ag}$ variation.

The effect of the metal layer on the transmittance and reflection spectra of the transparent contact, compared to the outer dielectric, which acts as a waveguide and has a surrounding effect, shows itself in the variation of the colour coordinates depending on the thickness. The variation of the colour coordinates of the MoO_3_/Ag/WO_3_ (10/${d}_{Ag}$/${d}_{{WO}_{3}}$ nm) transparent contact structure concerning ${d}_{Ag}$ and ${d}_{{WO}_{3}}$ are given in the CIE 1931 chromaticity diagram in Fig. 5a and b, respectively. The colour coordinates of the transparent contact with the highest AVT designed at ${d}_{Ag}$= 4 nm and ${d}_{{WO}_{3}}$= 6 nm are CIE x = 0.3110 and CIE y = 0.3271. They are quite close to the colour coordinates of achromatic point (${x}_{ap}$=0.3333, ${y}_{ap}$=0.3333), AM1.5G (${x}_{AM1.5G}$=0.3202, ${y}_{AM1.5G}$=0.3324) and D65 (${x}_{D65}$=0.3128, ${y}_{D65}$=0.3290). This superior performance, obtained in the colour characteristic of the MoO_3_/Ag/WO_3_ (10/4/6 nm) transparent contact structure with the highest AVT, increases the structure's potential to be used, especially in window applications that require neutral colour and in LED technologies.

With the increase of ${d}_{Ag}$ from 2 to 20 nm in the MoO_3_/Ag/WO_3_ (10/${d}_{Ag}$/6 nm) transparent contact structure, The colour coordinates shifted from the achromatic point to the blue region along Planckian locus. CIE x decreased by 23.43% from 0.3154 to 0.2415, and CIE y decreased by 25.12% from 0.3299 to 0.2470. The blueshift is due to a serious decrease in the wavelengths responsible for the red colour and the IR region with the increase of ${d}_{Ag}$, especially in the transmittance spectrum of the transparent contact structure. The effect of ${d}_{{WO}_{3}}$ on colour coordinates is less than that of ${d}_{Ag}$. This comparison can be better understood by examining the transmittance spectra given in Fig. 3b. With the increase of ${d}_{Ag}$, the transmittance decreases, especially in the region where the colour matching functions are responsible for red, while the spectra of the transmittance spectra with ${d}_{{WO}_{3}}$ change relatively at the same rate for all colours.

In determining the optimal conditions for the MoO_3_/Ag/WO_3_ transparent contact, the adjustment of ${d}_{Ag}$ should be made by evaluating the colour coordinates and AVTs together since the metal layer has a limiting effect on the AVT. However, even for ${d}_{Ag}$=20 nm, the AVT of the transparent contact is 36.24%. This value is greater than 25%, considered the maximum limit for window applications. Therefore, this feature indicates that colour modification can be achieved with a metal layer in the MoO_3_/Ag/WO_3_ (10/${d}_{Ag}$/6 nm) transparent contact structure. Especially for an LED that emits light in a different colour, integrating the MoO_3_/Ag/WO_3_ transparent contact into the structure with convenient design parameters can change the colour of the light emitted by the LED. In particular, the shift to blue offers significant potential for blue LED technology. In addition, the fact that the outer dielectric does not seriously affect the colour coordinates requires ${d}_{{WO}_{3}}$ to be considered an effective parameter only in the evaluation of AVT.

### Evaluation on CRI of MoO_3_/Ag/WO_3_ structure

**Figure 6:**
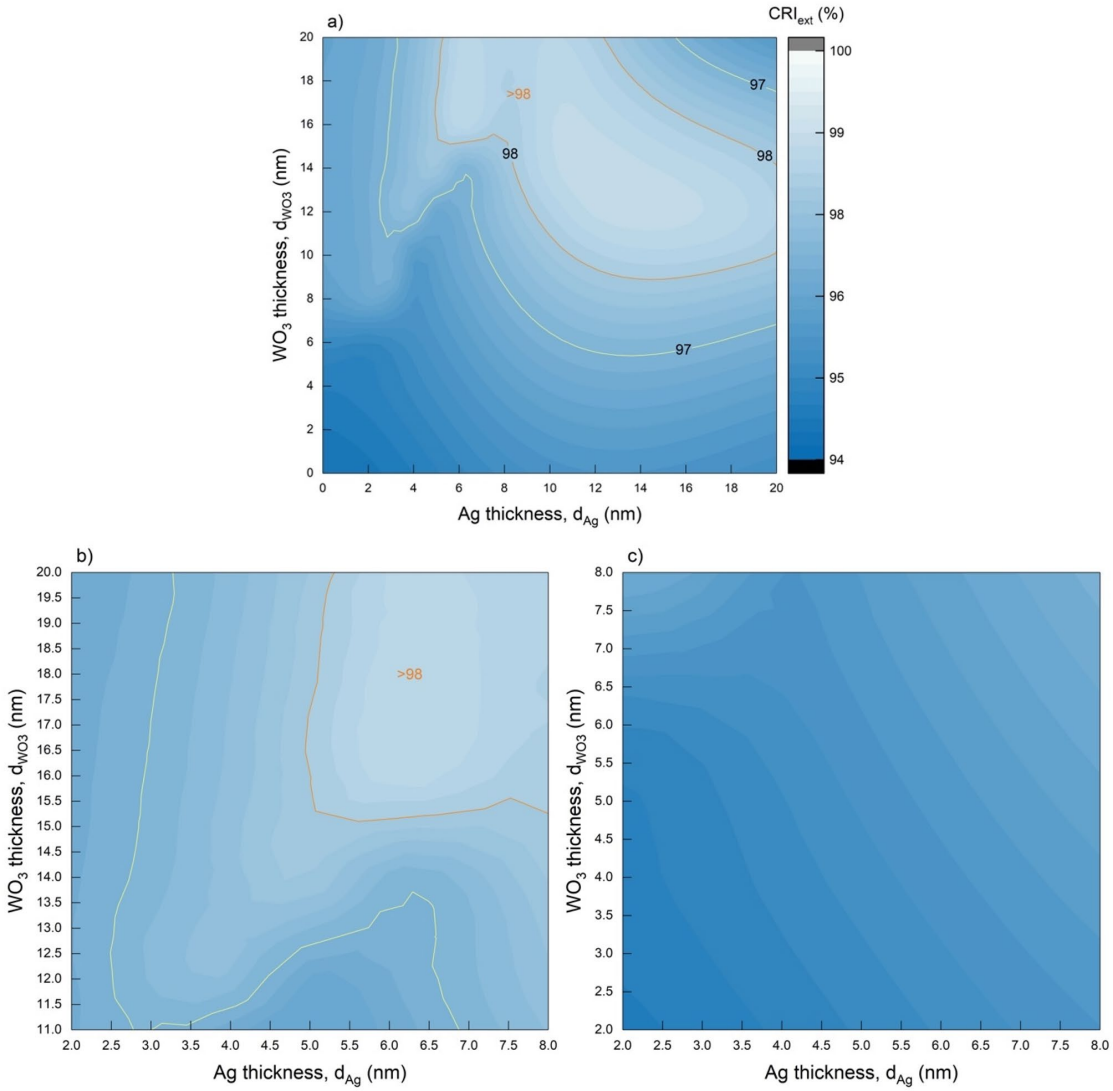
CRI_ext_ mapping of transparent contact structure depending on thickness. Distribution of CRI_ext_ values corresponding to $d_{Ag}$ and $d_{{WO_{3} }}$ for MoO_3_/Ag/WO_3_ (10/$d_{Ag}$/$d_{{WO_{3} }}$ nm) in the range of (**a**) 0 < $d_{Ag}$, $d_{{WO_{3} }}$ < 20 nm, (**b**) 11 < $d_{{WO_{3} }}$ < 20 nm and (**c**) 2 < $d_{{WO_{3} }}$ < 8 nm. Areas with orange and yellow straight-line contours contain CRI_ext_ values greater than 98% and 97%, respectively.

It is not sufficient for all optoelectronic devices to examine the characteristic of MoO_3_/Ag/WO_3_ transparent contact only in terms of AVT and colour coordinates and determine the optimal structure parameters. It is also essential to evaluate CRIs for optoelectronic devices such as LEDs, specially designed to serve lighting and high-resolution imaging technology. The ability of a light source to accurately reproduce the colours of an illuminated object is determined by the CRI, and values above 90 are classified as excellent. CRIs of 80 and above are acceptable for optoelectronic devices for indoor and commercial applications. However, it is crucial to obtain CRIs of 90 and above, especially for the exhibition and window lighting, screen display technologies where colour appearance is essential. The distribution of CRI_ext_ values for MoO_3_/Ag/WO_3_ (10/${d}_{Ag}$/${d}_{{WO}_{3}}$ nm) calculated in the 0–20 nm range of ${d}_{Ag}$ and ${d}_{{WO}_{3}}$ using the optical characteristics obtained by TMM is given in Fig. 6.

AVT, CRI_ext_, CIE x and y, CCT, T_max_ and ${\lambda }_{max}^{T}$ values of MoO_3_/Ag/WO_3_ transparent contact structure selected according to the variation of CRI_ext_ values and their maximum-minimum status are given in Table 1b. The CRI_ext_ values of the MoO_3_/Ag/WO_3_ (10/${d}_{Ag}$/${d}_{{WO}_{3}}$ nm) transparent contact structure are above 94 for ${d}_{Ag}$ and ${d}_{{WO}_{3}}$ in the range of 0–20 nm. This characteristic shows that the transparent contact structure has an excellent colour rendering property. Especially for ${d}_{Ag}$>5 nm and ${d}_{{WO}_{3}}$>15 nm, CRI_ext_ takes values greater than 98. In the region of 7 > ${d}_{Ag}$>5.5 nm and 20 > ${d}_{{WO}_{3}}$>15.5 nm, CRI_ext_ has a maximum value of 98.6. AVT, CRI_ext_, CIE x and y, CCT, ${T}_{max}$ and ${\lambda }_{max}^{T}$ values of MoO_3_/Ag/WO_3_ transparent contact structure selected according to the variation of CRI_ext_ values and their maximum-minimum status are given in Table 1b.

The CRI_ext_ of the MoO_3_/Ag/WO_3_ transparent contact structure, which is designed based on the highest AVT and neutral colour coordinates in the ${d}_{Ag}$=4 nm and ${d}_{{WO}_{3}}$=6 nm parameters, is 95.75% and is not at its maximum value. Therefore, the optimal structure (${d}_{Ag}$=4 nm, ${d}_{{WO}_{3}}$=6 nm) determined based on AVT and neutral colour values may not show high performance when evaluated in terms of CRI_ext_. Therefore, evaluating the AVT given in Fig. 2 and the CRI_ext_ distributions given in Fig. 6 together provides a more optimal structure.

When Fig. 3 and Table 1b are examined, it is evident that a thick outer dielectric layer is required to achieve high CRI_ext_ values. In this case, when the AVT distribution given in Fig. 2 is examined, $d_{Ag}$ should be in the range of 2–7 nm for high $d_{{WO_{3} }}$ values. With a more detailed analysis, it is understood that $d_{Ag}$ = 6 nm and $d_{{WO_{3} }}$ = 16 nm for both AVT and CRI_ext_ and neutral colour. For $d_{Ag}$ = 6 nm and $d_{{WO_{3} }}$ = 16 nm, the AVT is 95.38%, the CRI_ext_ is maximum 98.58, and the CIE x and y colour coordinates are 0.3168 and 0.3350, respectively. In addition, in these structure parameters, $\lambda_{max}^{T}$ is very close to 550 nm and has a value of 582.9 nm. Considering that commercial LEDs have reached 98 CRI today, with the achieved CRI of 98.58 and TCS09 of 94.80, MoO_3_/Ag/WO_3_ transparent contact for $d_{Ag}$ = 6 nm and $d_{{WO_{3} }}$ = 16 has the potential to be highly applicable both as a transparent contact structure and conductive sheath for existing commercial LEDs and imaging technologies provided with them.

**Figure 7:**
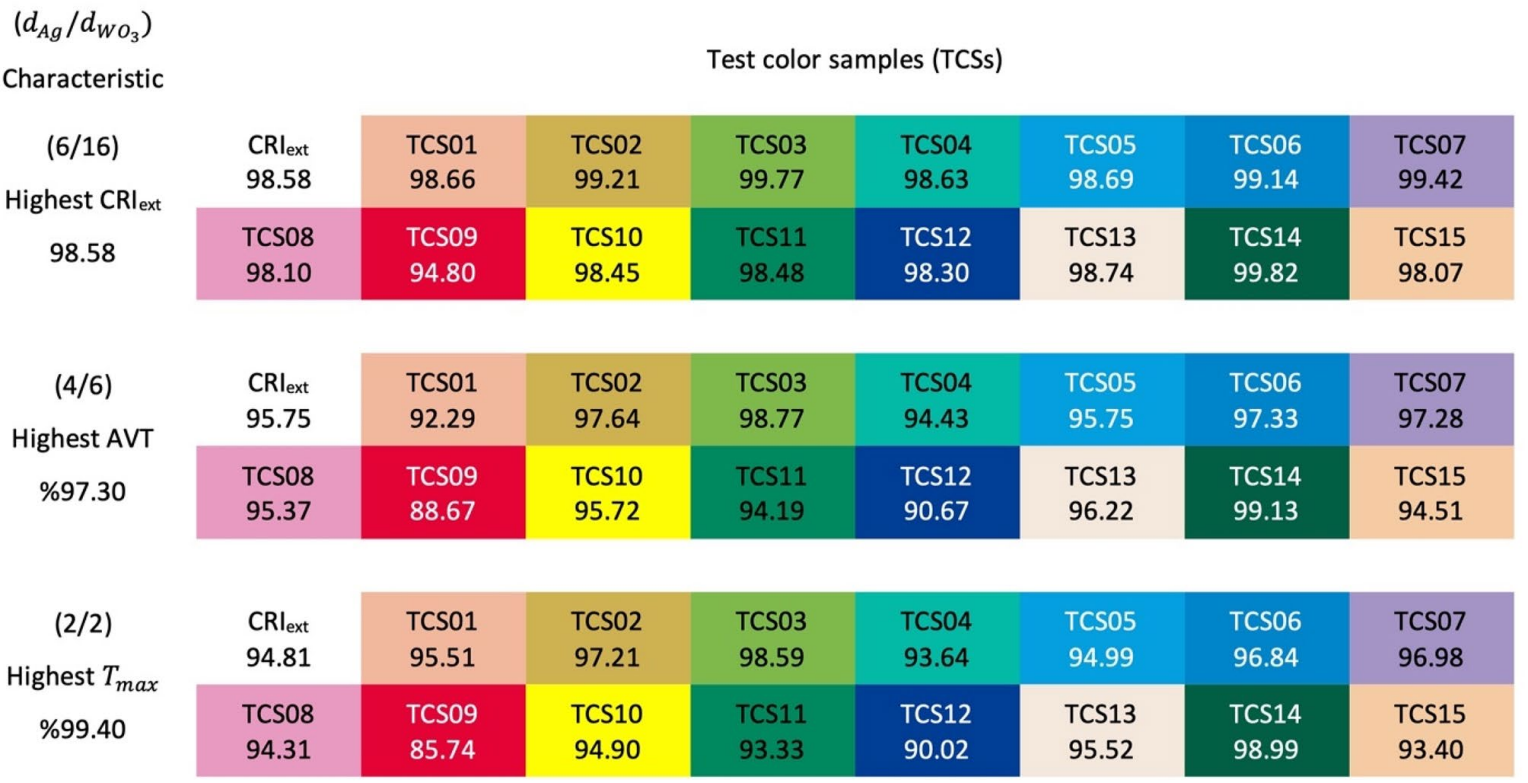
TCS classification of transparent contact structures. CRI for each test colour sample of MoO_3_/Ag/WO_3_ (10/$d_{Ag}$/$d_{{WO_{3} }}$ nm) transparent contact structure with the highest AVT ($d_{Ag}$ = 4 nm, $d_{{WO_{3} }}$ = 6 nm), highest CRI_ext_ ($d_{Ag}$ = 6 nm, $d_{{WO_{3} }}$ = 16 nm) and highest transmittance ($d_{Ag}$ = 2 nm, $d_{{WO_{3} }}$ = 2 nm).

In addition to the CRI_ext_ evaluation, it is essential to know how the colour renderings of the optimally determined structures are for each test colour sample (TCS). TCS analysis determines which transparent contact structure to be used in various applications can give specific standard colour with which rendering and how faithfully it is. Within the scope of the study, colour renders analysis was performed by making calculations over 15 TCS for the MoO_3_/Ag/WO_3_ transparent contact structure. The TCS values for the MoO_3_/Ag/WO_3_ transparent contact structure with the highest AVT ($d_{Ag}$ = 4 nm, $d_{{WO_{3} }}$ = 6 nm), the highest CRI_ext_ $(d_{Ag}$ = 6 nm, $d_{{WO_{3} }}$ = 16 nm), and the highest transparency ($d_{Ag}$ = 2 nm, $d_{{WO_{3} }}$ = 2 nm) are given in Fig. 7.

As expected for the structure with optimal values of $d_{Ag}$ = 6 nm and $d_{{WO_{3} }}$ = 16 nm based on CRI_ext_, there is a colour rendering index of over 90 for all TCSs. For structures designed based on AVT and T_max_, the values of all TCSs except TCS09 are pretty high and more significant than the perfection limit of 90. Therefore, the contact structure designed in these parameters is very suitable for optoelectronic applications such as lighting and display technologies that include specific colour applications. An electrical evaluation of MoO_3_/Ag/WO_3_ (10/6/16 nm), which is optimally presented for CRI, is also noteworthy in the literature. Especially, for $d_{Ag}$ > 4 nm, the $R_{sh}$ decreases and becomes around 4 ${\Omega }sq^{ - 1}$ in $d_{Ag}$ = 6 nm^53^. Therefore, the MoO_3_/Ag/WO_3_ (10/6/16 nm) also has a very convenient contact properties for electrical performance.

TC09 measures how well a light source or transparent structure can reproduce red. The red colour is critical in applications such as photography, textiles and the production of human colour tones. Many objects around us appear as a mixture of colours, including red. For example, skin tone is very sensitive to the red colour of blood flowing under the skin. With all these features and its proximity to daylight or incandescent bulbs, the TCS09 stands out as a special consideration among other TCSs. A system with a low TCS09 score will display red as far from its colour, or even green. Therefore, the TCS09 is significant for LEDs, which form the backbone of daily or professional lighting technology today.

In analysis, it is not easy to obtain TCS09 at a high value compared to other TCSs, especially for mathematical evaluation over spectra. In addition, the TCS09 value is highly dependent on the spectral characteristics of the device. Therefore, the TCS09 rating is classified as suitable for values of 50 and above and excellent for values of 90 and above, unlike the CRI or CRI_ext_ scale. TCS09 values for MoO_3_/Ag/WO_3_ transparent contact designed based on AVT and $T_{max}$ are quite high and are 88.67 and 85.74, respectively. For the structure designed based on CRI_ext_, the TCS09 metric is rated excellent and has a value of 94.80. Therefore, MoO_3_/Ag/WO_3_ (10/$d_{Ag}$/$d_{{WO_{3} }}$ nm) transparent contact structure for $d_{Ag}$ = 6 nm and $d_{{WO_{3} }}$ = 16 nm parameters is a high-performance contact system that can be integrated into optoelectronic devices based on LED lighting applications and imaging technologies.

When the $d_{Ag}$ are examined for the optimal structures presented in Fig. 7, it is remarkable that ultra-thin metal layer such as 2 nm, 4 nm and 6 nm is optimally presented. In practice, properties such as roughness and thickness of ultra-thin Ag films can significantly degrade the resistivity and sheet resistance. This is due to the formation process of the Ag layer, especially in physical vapor deposition techniques. Because it is usual for Ag particles to accumulate in islands during the deposition process. This requires the investigation of different transport mechanisms for the movement of electrons in the metal layer. Therefore, using various techniques such as sputtering and electron beam evaporation in the literature and improving the deposition parameters in these techniques, ultra-thin, ultra-smooth, continuous, low-loss and low-$R_{sh}$ Ag thin films can be produced^53,54^. Especially in the sputter system, by increasing the sputtering time for Ag and narrowing the gap between Ag islands, $R_{sh}$ for 4 nm Ag thin-film could be reduced considerably^53^. In addition, with the electron beam evaporation system, the root mean square roughness values could be improved from 0.73 nm to 0.22 nm even with the thinning of the thickness from 6 to 1 nm for ultra-thin Ag^54^. With all these developments and improvements in different deposition techniques for the production of ultra-fine Ags, the optimal structures presented in the study have the potential to be produced and offer good electrical properties.

### Evaluation on colour Coordinates of MoO_3_/Ag/WO_3_ structure

**Figure 8:**
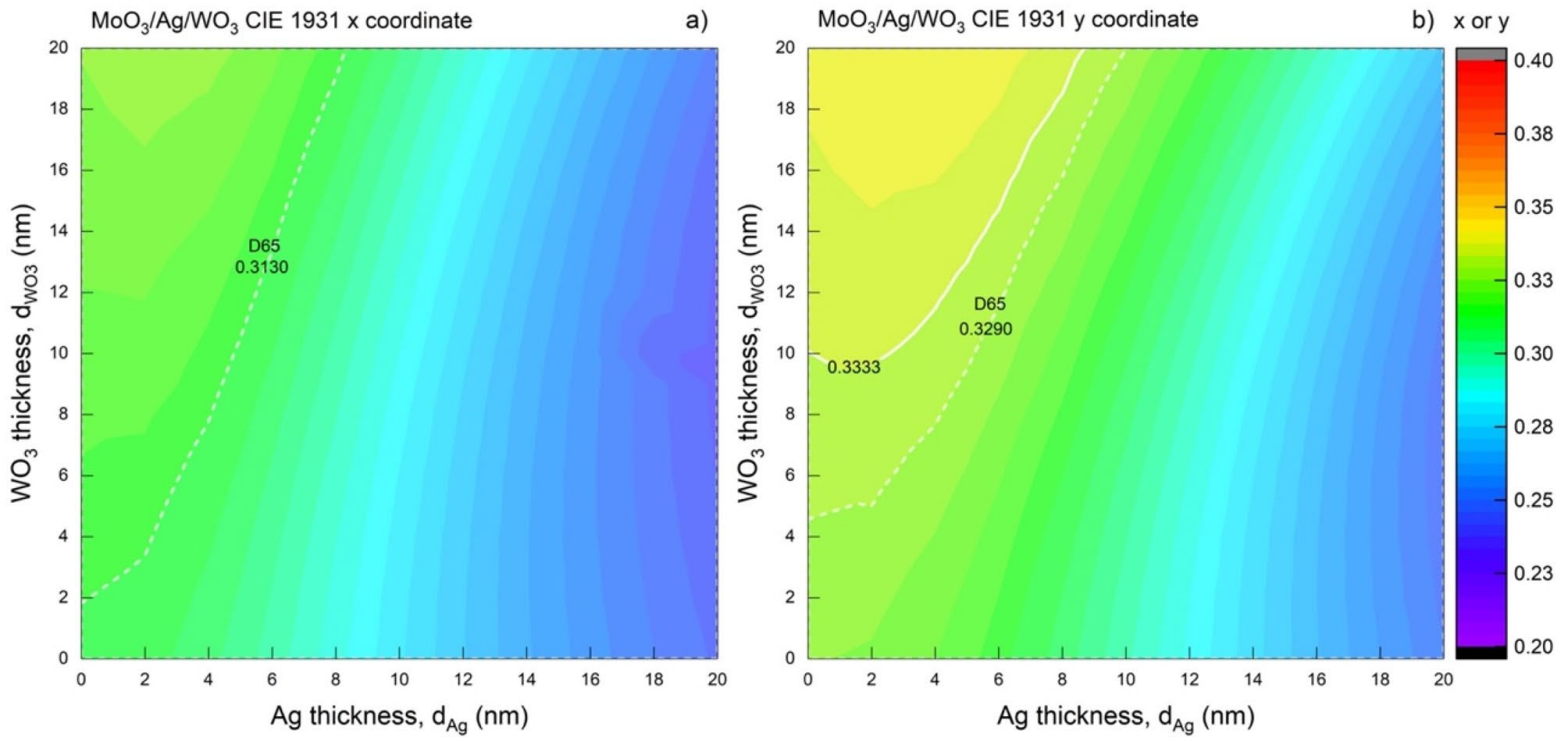
Mapping of the CIE 1931 colour coordinates of the transparent contact structure depending on the thickness. The distribution of (**a**) CIE x and, (**b**) y colour coordinates corresponding to the $d_{Ag}$ and $d_{{WO_{3} }}$ values for MoO_3_/Ag/WO_3_ (10/$d_{Ag}$/$d_{{WO_{3} }}$ nm). The dashed and solid white lines belong to the contours representing the colour coordinate of the D65 and achromatic point, respectively.

The colour perception and CRI_ext_ characteristics are also imperative to examine the colour coordinates of the MoO_3_/Ag/WO_3_ according to the $d_{Ag}$ and $d_{{WO_{3} }}$ changes. The distribution of colour coordinates obtained from optical characteristics by TMM for MoO_3_/Ag/WO_3_ (10/$d_{Ag}$/$d_{{WO_{3} }}$ nm) according to $d_{Ag}$ and $d_{{WO_{3} }}$ was studied on. For $d_{Ag}$ and $d_{{WO_{3} }}$ in the range of 0–20 nm is represented in Fig. 8.

**Figure 9:**
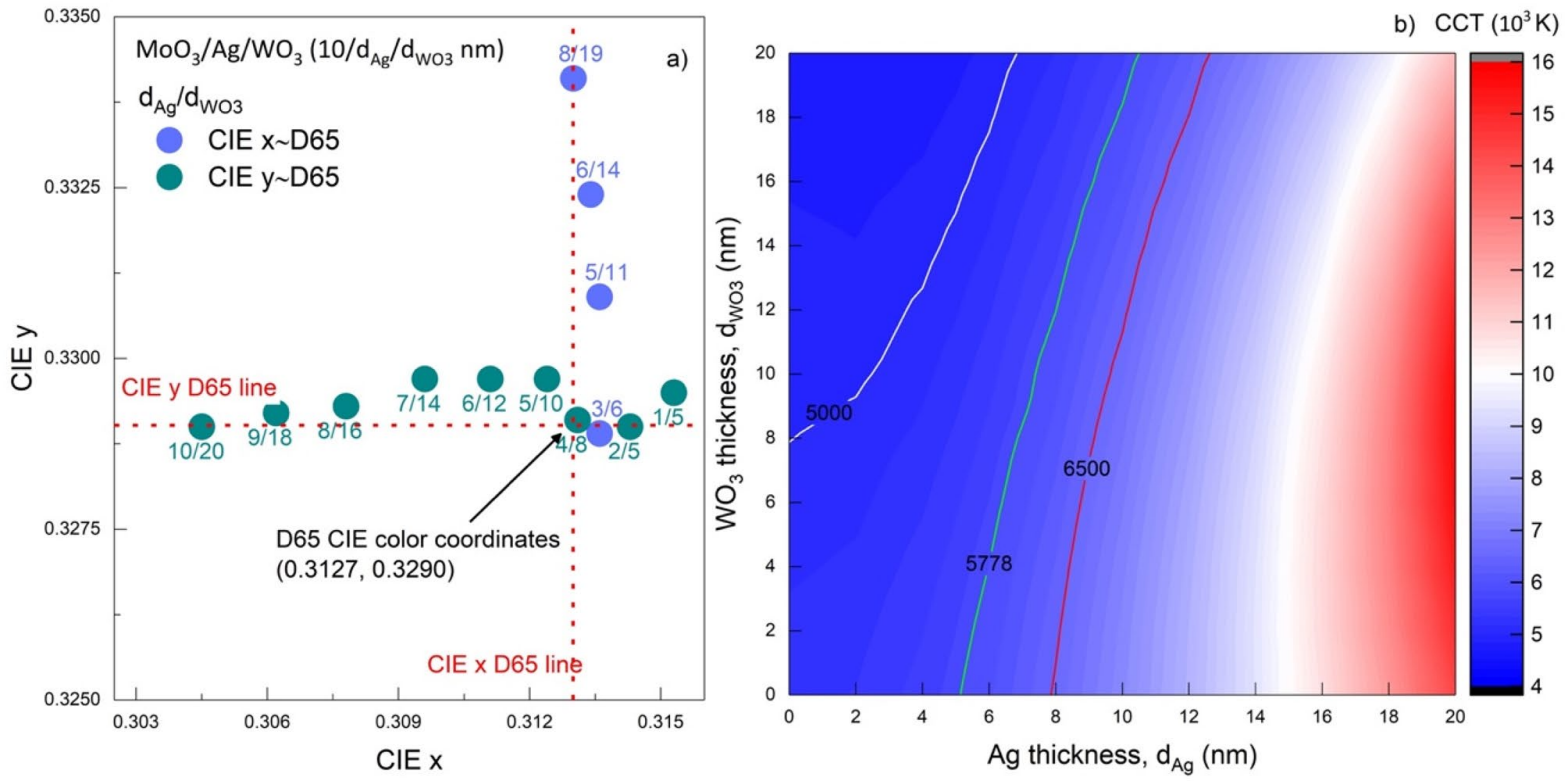
Colour properties of the transparent contact structure with a CIE x and y on D65. (**a**) The colour coordinates' change of the structures corresponding to the exact values of $d_{Ag}$ and $d_{{WO_{3} }}$ dependently the case that the CIE x and y colour coordinates of the MoO_3_/Ag/WO_3_ (10/$d_{Ag}$/$d_{{WO_{3} }}$ nm) transparent contact are on the D65 point. (**b**) Mapping the CCT variation of the structure considering $d_{Ag}$ and $d_{{WO_{3} }}$ values for MoO_3_/Ag/WO_3_ (10/$d_{Ag}$/$d_{{WO_{3} }}$ nm). The white, green, and red-lined contours refer to the CCT values of 5000 K, 5778 K, and 6500 K, respectively.

As mentioned in the AVT characteristic, examining the Fig. 8, it is seen that the metal layer is more effective than the outer dielectric on the colour coordinates. Also, the colour coordinates dependent on $d_{Ag}$ decrease after 7 nm for CIE x and 9 nm for CIE y. The obtainment colour coordinates very close to D65 and the achromatic point is possible for a wide range of $d_{Ag}$ and $d_{{WO_{3} }}$. On the other hand, the CIE x coordinate for the MoO_3_/Ag/WO_3_ (10/$d_{Ag}$/$d_{{WO_{3} }}$ nm) contact cannot be at the Planckian locus value, but this is possible for the CIE y. A structure with colour characteristics of D65 coordinate can also be stated for MoO_3_/Ag/WO_3_ (10/$d_{Ag}$/$d_{{WO_{3} }}$ nm), as seen from the D65 contour line in Fig. 8a and b. The optical parameters of the structures corresponding to the exact values ​​of $d_{Ag}$ and $d_{{WO_{3} }}$ are noted in Tables 2a and b. In addition, the change of colour coordinates and their relations with each other and together with $d_{Ag}$ and $d_{{WO_{3} }}$ are given in Fig. 9a.

**Table 2:** Optical parameters of transparent contact structure with colour over D65.

(a)					(b)				
$d_{Ag}$/$d_{{WO_{3} }}$	${\varvec{x}}\sim$ **D65**	$y$	AVT(%)	CRI_ext_	$d_{Ag}$/$d_{{WO_{3} }}$	$x$	${\varvec{y}}\sim$ **D65**	AVT(%)	CRI_ext_
8/19	**0.3130**	0.3341	94.19	98.18	10/20	0.3045	**0.3290**	91.21	98.44
6/14	**0.3134**	0.3324	95.89	97.01	9/18	0.3062	**0.3292**	92.78	98.44
5/11	**0.3136**	0.3309	96.5	96.37	8/16	0.3078	**0.3293**	94.12	98.03
**4/8**	**0.3131**	**0.3291**	96.97	95.76	7/14	0.3096	**0.3297**	95.22	97.42
3/6	**0.3136**	0.3289	97.016	95.32	6/12	0.3111	**0.3297**	96.07	96.82
					5/10	0.3124	**0.3297**	96.66	96.27
					**4/8**	**0.3131**	**0.3291**	96.97	95.76
					2/5	0.3143	**0.3290**	96.22	95.04
					1/5	0.3153	**0.3295**	94.97	94.92

Optical parameters of the structures corresponding to the exact values of $d_{Ag}$ and $d_{{WO_{3} }}$ in the case that (a) CIE x and, (b) y coordinates are at D65.

Significant values are in [bold].

As $d_{Ag}$ and $d_{{WO_{3} }}$ decrease together in structures where the CIE x is equal to D65's x value, the CIE y coordinate also approaches its value in the D65 line. The same behaviour is obtained for structures where the coordinate is equal to D65. With this examination, as shown in Fig. 9a, the colour coordinate of the MoO_3_/Ag/WO_3_ transparent contact structure designed for $d_{Ag}$ nm and $d_{{WO_{3} }}$ = 8 nm is equal to the D65 colour coordinates. In addition, the AVT and CRI_ext_ values for this structure are pretty high, with values of 96.97% and 95.76%, respectively.

The colour coordinates equal to D65 are the characteristic properties that make the current structure suitable for use in various optoelectronic applications, especially acting as a light source. Moreover, CCTs are noteworthy in optoelectronic devices designed for lighting technology, various light sources and illuminations closest to daylight. Here, CCTs were calculated from the optical characteristics of the MoO_3_/Ag/WO_3_ transparent contact structure obtained by TMM. The distribution of the values corresponding to $d_{Ag}$ and $d_{{WO_{3} }}$ in the 0–20 nm range is given in Fig. 9b.

CCTs in the range of 5000–6500 K for MoO_3_/Ag/WO_3_ can be obtained for a wide value range of $d_{Ag}$ and $d_{{WO_{3} }}$. This characteristic shows that CCT is not limiting in optimising the MoO_3_/Ag/WO_3_ transparent contact. It can act as an illuminator quite close to daylight.

## Method

### Calculation of optic spectrum

The design of DMDs, which are transparent top contacts with MoO_3_/Ag/WO_3_ structure, was carried out by calculating the absorption, transmittance, reflection spectra and evaluating the thickness parameters. The transfer matrix method (TMM), a very effective one used in the simulations of optoelectronic devices, was applied to perform the calculations. It is the most potent and widely used method to analyze how the electromagnetic wave propagates in the structure and to determine the optical characteristics of the structure theoretically, especially in structures such as DMD, where different dielectric layers are grown on top of each other^10^. The method followed, and the equations used in calculating made with TMM are given in detail in our previous study^10^ and Supplementary Information.

### Calculation of average visible transmittance

AVTs evaluate the transparency properties of contact systems and identify the transmittance characteristic in the visible light wavelength range (370–740 nm), taking into account the photonic response of the human eye. The transmittance spectra of the MoO_3_/Ag/WO_3_ system calculated by TMM were employed when making AVT calculations. The method followed, and the equations used in calculating AVTs are given in detail in our previous study^10^ and Supplementary Information.

### Calculation of CIE 1931 colour coordinates

Another significant characteristic of transparent contact structures is the colour coordinates in the CIE 1931 chromaticity diagram (CIE x and y) as vital as AVT. Often used to determine the colour properties of transparent optoelectronic devices, the design of this diagram is based on the photonic response of the human eye. The method followed, and the equations used in calculating the CIE 1931 colour coordinates of the MoO_3_/Ag/WO_3_ transparent contact system are given in detail in our previous study^10^ and Supplementary Information.

### Calculation of colour render ındex

Colour render index (CRI) measures how precise the colour of an object is when it is illuminated with an ideal or natural light source^22^. CRIs of 90 and above are considered excellent, while CRIs below 80 is generally considered poor^40^. Therefore, CRI creates a measurement system that gives whether the colours of illuminated objects are true to their originality and takes values in the range of 0–100. A CRI of 100 can be seen in standardised daylight sources or incandescent lamps. The method followed, and the equations used in calculating the CRIs of the MoO_3_/Ag/WO_3_ transparent contact system are given in detail in our previous study^10^ and Supplementary Information.

### Calculation of correlated colour temperature

One key criterion of defining predominantly white light sources is that the associated colour temperature (CCT), a complete, one-dimensional measurement system, identifies a particular point along the blackbody curve on the CIE 1931 chromaticity diagram. CCT also emerges as a system that shows the similarity of optoelectronic devices with transparent or semi-transparent characteristics to light emitters in various technological applications. The method followed, and the equations used in calculating the CCTs of the MoO_3_/Ag/WO_3_ transparent contact system are given in detail in the Supplementary Information.

## Supplementary Information

Supplementary Information.

## Data Availability

The datasets used and/or analysed during the current study available from the corresponding author on reasonable request.
